# Cultivating hope: reframing the ‘heart-sink’ consultation

**DOI:** 10.3399/BJGP.2025.0728

**Published:** 2026-02-01

**Authors:** Mohammed Mustafa, Hassan Awan, Awadalla Youssef, Carolyn A Chew-Graham

**Affiliations:** 1 School of Medicine, Cardiff University, Cardiff, UK; 2 School of Medicine, Keele University, Keele, UK; 3 Azhari Consultancy and Training, Manchester, UK

General practice increasingly operates within an environment of scarcity — of time, appointments, and often of hope. Rising workload, workforce crises, and growing multimorbidity have stretched the therapeutic reach of primary care to its limits.^1^ Many GPs recognise the recurring heart-sink consultation: a term first used to describe patients who evoke a sense of helplessness or dread in their doctors.^2^ Later reflection recognised that such encounters often arise when clinicians feel powerless to meet complex needs.^3^ Such moments test both clinical skill and morale, and can be a cause of clinician burnout. Yet evidence suggests that *hope* — defined, measurable, and teachable — can itself be a therapeutic intervention. Hope is not sentimental optimism. Snyder described it as the combination of *agency* (belief in one’s capacity to act) and *pathways* (belief that routes to goals exist).^4^

## Hope as an evidence-based construct

Evidence links hope to better psychological adjustment and self-management among people living with long-term conditions.^5,6^ In people with pain, higher hope and optimism predict less distress and improved functioning.^7^ A systematic review of hope-fostering interventions in palliative care further indicates potential improvements, including reduced depression in palliative populations.^8^ Cultivating hope is therefore an active process that sustains motivation when the possibility of cure is uncertain, positioning it as *integral*, not peripheral, to evidence-based care.

## From despair to hope-centred dialogue

If despair defines the heart-sink encounter, fostering a shared sense of hope may redefine what counts as effective care. Hope can be cultivated in ordinary consultations, not as false reassurance but as a realistic orientation to what remains possible. Empathic, goal-directed dialogue sustains motivation even when cure is impossible. Clinicians who respond constructively to patient emotion improve communication quality without extending consultation length.^9^ Such ‘micro-interventions’ do not incur financial cost, but depend on reflective capacity and emotional bandwidth — resources as finite as time itself — yet they fit naturally within the GP 10-minute consultation.

## Hope in context and equity

Qualitative research among South Asian men with long-term conditions highlights that distress often arises from social disconnection and stigma rather than disease severity.^10,11^ Participants described drawing strength from faith, family, and community — sources of hope that can be explored in consultations. Recognising and validating these meaning-making resources can enhance engagement and outcomes, particularly for patients facing structural disadvantage. When services overlook such sources, opportunities for partnership are lost. In this sense, attention to hope also supports equity — enabling patients to regain a measure of agency within constrained systems.

*“Reframing the ‘heart-sink’ encounter as a chance to foster hope can restore a sense of therapeutic competence and purpose.”*

## When hope heals the healer

Burnout threatens both clinicians and patient safety. Reframing the ‘heart-sink’ encounter as a chance to foster hope can restore a sense of therapeutic competence and purpose. For clinicians, erosion of professional meaning and perceived efficacy are closely linked to burnout and career disengagement. A recent study of UK GPs found that emotional exhaustion and reduced accomplishment predicted intentions to leave practice.^1^ Restoring a sense of purpose and therapeutic agency is therefore central to sustaining hope within the workforce. Each small act of enabling progress — a realistic goal agreed, a fear normalised, a strength recognised — affirms that care still matters, for both patient and clinician alike. Interventions that enhance positive affect and meaning have been shown to improve clinician wellbeing and professional satisfaction as well as reduce depressive symptoms in patients.^8,9^ Hope, therefore, is not only something we give; it is something we *share*.

## Translating hope into practice

Translating hope into everyday consultations can build on established communication principles from models such as Calgary-Cambridge^12^ and motivational interviewing,^13^ as well as on emerging frameworks such as Irshaad Tazkiyat Care (ITC). ITC, which underpins our ongoing study in Wales, adapts these principles within a hope-centred and ethically grounded approach to care. Evidence shows that empathic recognition of emotion, validation, and collaborative goal-setting improve both patient satisfaction and clinician wellbeing.⁹ The ‘name, normalise, next step’ sequence synthesises these techniques into a concise, teachable heuristic. It offers a structured way to integrate hope-centred dialogue into routine consultations and is currently being tested for feasibility and acceptability.

Name: identify the patient’s core concern or value — *‘It sounds like walking to the shop again would be a big step for you.’* Normalise: validate and contextualise — *‘Many people find progress slower than they hoped; that’s normal.’* Next step: co-develop a realistic action according to the patient’s preferences — *‘Let’s plan one way to start moving toward that goal.’*

Such brief, structured dialogue reinforces both agency and pathways — the twin components of hope — without extending consultation time. Progress can then be tracked in what matters to patients: walking to the shop, caring for a grandchild, returning to prayer.

## Leadership and morale

Hope also operates at team level. Compassionate, distributed leadership — enabling autonomy, noticing improvement, celebrating small wins — builds morale and retention more effectively than exhortation alone.^14^ Leaders who model hope make it permissible for clinicians to believe again in the value of their everyday work.

## Restoring hope within health care

Reframing hope as a clinical skill rather than a sentimental ideal restores meaning to medicine’s most constrained spaces. For patients, it nurtures agency; for clinicians, it restores purpose; for leaders, it protects culture and morale. For educators, it invites us to teach language and presence as therapeutic tools. For researchers, it demands that we measure not only what is cured but also what is restored. In an NHS short of almost everything, hope remains one resource we can still afford to give — and to keep.
